# Leveraging Multi-Source Data Fusion Approach for Fine-Grained Affective-Appraisal Analysis in TPD-Oriented Online Professional Learning

**DOI:** 10.3390/bs16061025

**Published:** 2026-06-18

**Authors:** Di Chen, Xinyue Xu, Ruiyang Gao, Yuhong Liu

**Affiliations:** Faculty of Artificial Intelligence in Education, Central China Normal University, Wuhan 430079, China; chendi@mails.ccnu.edu.cn (D.C.); xinyue_xu@mails.ccnu.edu.cn (X.X.); jiaqin_wan@mails.ccnu.edu.cn (Y.L.)

**Keywords:** pre-service teacher professional learning, teacher professional development, fine-grained affective-appraisal analysis, affective computing, multi-source data fusion

## Abstract

Teacher professional development (TPD) is increasingly mediated by online platforms, yet emotion analysis in this context remains underdeveloped because teachers’ professional discourse is often reflective, evaluative, and shaped by professional norms. To address this challenge, this study proposes a fine-grained, low-intrusion affective-appraisal analysis framework for TPD-oriented online professional learning that integrates textual evidence with platform interaction logs. The framework retains pleasure, arousal, and dominance from the pleasure–arousal–dominance (PAD) model and introduces utility as an appraisal-related dimension, capturing teachers’ perceived usefulness, value judgment, and professional learning gain. Methodologically, it combines textual representations based on Bidirectional Encoder Representations from Transformers (BERT), intra-week long short-term memory (LSTM) aggregation, interpretable behavioral-log features, and feature-level fusion. Data were collected from an authentic TPD-oriented online course involving 107 pre-service teachers, yielding 1276 teacher-week samples from 4300 texts and 264,028 interaction records. Results show that intra-week sequential modeling improves the macro-averaged F1 score (Macro-F1) over both the term frequency–inverse document frequency plus support vector machine (TF-IDF+SVM) baseline and BERT-based weekly text concatenation, with statistically significant gains over the non-sequential BERT-concat model across all four dimensions. Adding interaction logs improves accuracy across all dimensions and provides complementary process-based evidence, especially for arousal and utility. By linking a four-dimensional affective-appraisal framework with text-log fusion, this study offers a scalable and context-sensitive approach to affective-appraisal analytics in pre-service teacher professional learning.

## 1. Introduction

Teacher professional development (TPD) is widely regarded as a central mechanism for strengthening instructional quality and supporting educational change (Morina et al., 2025; Meyer et al., 2023). As online and platform-mediated formats expand, researchers increasingly attend not only to participation and outcomes but also to teachers’ emotional experiences during professional learning (Ehlert et al., 2025). Emotions influence teachers’ engagement, persistence, reflection, and identity construction during professional development (Saunders, 2013; Gaines et al., 2019). These emotional processes are difficult to observe directly because professional discourse is typically reflective, evaluative, and shaped by institutional and professional norms (Stark & Bettini, 2021). This issue is especially salient in online professional development communities, where emotional experience can shape teachers’ sense of belonging and sustained participation (Nash, 2022).

Existing approaches remain insufficient in three respects. Self-report measures provide valuable subjective evidence, but they are ill-suited to fine-grained, continuous, and process-sensitive monitoring (Taxer & Gross, 2018). Many computational studies still rely on coarse positive–negative categories or text-only pipelines, which makes it difficult to capture the mixed, low-intensity, and value-laden emotions common in TPD discourse (Shaik et al., 2023). Recent reviews of automated emotion recognition in online learning likewise show that progress in modeling does not eliminate the need for interpretable task definitions and context-sensitive evidence (Yu et al., 2024). Meanwhile, multimodal emotion recognition has advanced quickly, but many approaches depend on richer data integration strategies that complicate large-scale deployment on authentic professional development platforms (Mu et al., 2020). This difficulty becomes even more pronounced when multimodal systems are evaluated in terms of governance, implementation complexity, and real-world sustainability (Mohammadi et al., 2025).

To address this challenge, this study proposes a fine-grained affective-appraisal analysis framework tailored to TPD-oriented online professional learning by integrating textual data with interaction behavior logs. Conceptually, the framework extends the pleasure–arousal–dominance (PAD) model (Mehrabian, 1996) by integrating utility with pleasure, arousal, and dominance. While PAD captures affective valence, activation, and perceived control, utility functions as an appraisal-related dimension that represents perceived usefulness, value judgment, and sense of professional gain in professional learning contexts. The study pursues three objectives: first, to develop and operationalize a four-dimensional affective-appraisal representation for analyzing affective and appraisal-related cues in professional learning texts; second, to evaluate whether intra-week sequential aggregation of textual data improves week-level recognition compared with conventional and non-sequential text-based approaches; and third, to examine whether platform interaction logs provide complementary behavioral evidence for affective-appraisal recognition. Methodologically, the framework combines BERT-based textual representations, intra-week sequential aggregation, and interpretable features extracted from routine platform logs. Empirically, it is evaluated in an authentic TPD-oriented online course for pre-service teachers through comparisons among conventional text baselines, sequential text modeling, and text-log fusion. The study contributes a context-sensitive affective-appraisal representation and a scalable sensor-free fusion framework for week-level analysis of pre-service teachers’ professional learning.

## 2. Literature Review

### 2.1. Teacher Emotions in TPD-Oriented Online Professional Learning

Teacher emotion is not a peripheral aspect of teaching but a constitutive element of professional practice. Hargreaves (1998) conceptualized teaching as emotional practice embedded in institutional structures, interpersonal relationships, and moral expectations. Sutton and Wheatley (2003) further showed that teacher emotions shape motivation, cognition, instructional behavior, and classroom interaction. These perspectives indicate that emotions are not simply private psychological states; they are situated in professional roles, school cultures, and relationships with students, colleagues, and institutions.

In teacher professional development, emotions are closely tied to learning, reflection, and professional change. Saunders (2013) showed that reform and professional learning may evoke hope, anxiety, frustration, pride, and uncertainty, all of which influence teachers’ willingness to engage with new practices. Gaines et al. (2019) similarly highlighted that professional learning activities generate emotional experiences through task demands, peer interaction, self-efficacy judgments, and identity-related concerns. Teacher emotions are also shaped by display rules and normative expectations regarding what teachers should express in professional settings (Stark & Bettini, 2021). As a result, affective experience in professional learning is often indirect, restrained, and intertwined with reflection and evaluation.

Online and platform-mediated forms of professional learning add another layer of complexity. Informal online teacher communities and networks can support knowledge exchange, resource sharing, and identity development (Macià & García, 2016), whereas formally organized online communities vary in structure, participation patterns, and mechanisms of knowledge production (Lantz-Andersson et al., 2018). Course-based online professional development requires careful attention to interaction design, support structures, and practice-oriented tasks (Powell & Bodur, 2019). Platform affordances, community interaction, and sustained participation are central to professional learning quality and teacher engagement (Jin et al., 2024; Wang & Hu, 2025). Although flexibility and immediacy are important advantages of online professional learning, teachers may also experience time pressure, fragmented participation, and difficulties in translating learning into practice (Howard, 2021). These characteristics make emotional engagement important but difficult to observe directly.

The distinction between online communication and face-to-face professional interaction is particularly relevant for emotion analysis. In classroom teaching and in-person professional learning, emotional experience may be expressed through speech, gesture, facial expression, interpersonal timing, and immediate interaction. In online professional learning, by contrast, much of the available evidence is mediated through written reflections, discussion posts, resource access, and platform activity traces. Such evidence is more reflective, evaluative, and normatively filtered than real-time emotional expression. Consequently, online professional learning data should not be treated as a direct record of internal emotional states; rather, it can provide observable cues of affective orientation, perceived control, engagement, and value appraisal.

Recent research has linked online professional development to teacher-level and classroom-level outcomes (Meyer et al., 2023; Morina et al., 2025), and has shown that online formats can support participation and engagement, although sometimes at lower levels than face-to-face formats (Mulaimović et al., 2025). However, emotional variables are still often treated as ancillary outcomes, and the measurement of teacher emotion in professional development remains heavily dependent on questionnaires or qualitative evidence (Ehlert et al., 2025; Nash, 2022). These studies establish the relevance of emotion in professional learning while also pointing to the need for fine-grained, process-sensitive, and context-aware approaches to modeling affective experience in online environments.

### 2.2. Fine-Grained Affective-Appraisal Representation for Educational Settings

A central challenge in educational emotion research is how to represent affective experience with sufficient granularity while retaining interpretability and computational feasibility. Coarse polarity categories such as positive and negative are useful for broad sentiment classification, but they are poorly suited to educational settings in which learners or teachers may simultaneously experience difficulty, perceived value, engagement, uncertainty, and professional gain. This limitation is especially salient in professional learning, where written discourse often combines reflection, evaluation, and restrained affective expression.

One major tradition represents emotions as discrete categories. Control–Value Theory explains achievement emotions as arising from appraisals of control and value, thereby linking emotional experience to perceived competence, task importance, and outcome relevance (Pekrun, 2006). The Achievement Emotions Questionnaire further operationalizes multiple emotions such as enjoyment, pride, anxiety, boredom, and hopelessness across learning and performance contexts (Pekrun et al., 2011). Work on affective dynamics in learning has also shown that states such as confusion, engagement, and frustration evolve over time rather than remaining static (D’Mello & Graesser, 2012). These studies demonstrate the value of differentiating emotions, but discrete labels can be difficult to apply when professional learning texts contain weak, mixed, or evaluative expressions rather than explicit emotion words.

Dimensional theories provide a complementary route for fine-grained representation. Russell’s (1980) circumplex model describes affect in terms of valence and arousal, capturing both the pleasantness of experience and the level of activation. Mehrabian’s (1996) PAD framework adds dominance, which represents perceived control, agency, or influence in a given situation. In educational settings, these dimensions are useful because they distinguish between whether an experience is positive or negative, whether it is calm or activated, and whether the individual feels in control or constrained. Arousal is therefore not equivalent to positive or negative emotion; high arousal may correspond to enthusiasm, anxiety, anger, or urgent engagement, whereas low arousal may correspond to calmness, fatigue, or disengagement depending on the accompanying valence and dominance cues.

For TPD-oriented professional learning, affective representation also needs to account for value appraisal. Professional learning is inherently goal-directed: participants evaluate whether learning tasks, resources, discussions, and reflections are useful for teaching practice, professional growth, and future application. This aspect is theoretically consistent with Control–Value Theory, which positions value appraisal as a central antecedent of achievement emotions (Pekrun, 2006). It also aligns with the Technology Acceptance Model, in which perceived usefulness is defined as a key determinant of technology acceptance and continued use (Davis, 1989; Venkatesh & Davis, 2000). In educational technology research, perceived usefulness has been widely used to explain how teachers and learners evaluate digital learning environments and tools (Granić & Marangunić, 2019).

On this basis, utility can be conceptualized as an appraisal-related dimension that complements pleasure, arousal, and dominance. It does not function as a basic affective dimension in the same sense as PAD. Rather, it captures perceived usefulness, value judgment, and sense of professional gain, which may co-occur with but remain theoretically distinct from affective valence, activation, and perceived control. This distinction is important because professional learning may be experienced as demanding or frustrating while still being perceived as useful, and conversely, pleasant participation may not necessarily indicate high professional value.

Taken together, these perspectives support a profile-based representation in which pleasure, arousal, dominance, and utility jointly characterize affective and appraisal-related patterns in professional learning. This approach is useful for describing configurations in which emotional tone, activation, perceived control, and value appraisal may diverge, such as challenging but useful learning or low-salience participation. Framing the four-dimensional coordinates as interpretive profiles preserves the flexibility of dimensional modeling while remaining attentive to the situated and multifaceted nature of educational emotions.

### 2.3. Computational Affective-Appraisal Analysis in Online Professional Learning

Research on educational emotion has traditionally relied on self-report measures, interviews, diaries, observations, or physiological and behavioral measurements. Self-reports provide direct access to subjective experience, but they are limited in their capacity to capture fine-grained temporal variation and process-level dynamics (Taxer & Gross, 2018). In online professional learning, self-reports also depend on retrospective judgment and may be affected by social desirability or professional norms. These limitations do not make self-reports irrelevant, but they motivate the use of complementary process data that are naturally produced during learning activities.

Text-based emotion analysis offers one scalable source of evidence. Forum discussions, reflective journals, learning summaries, and course evaluations have been widely used to model affect, engagement, and learning experience in online education. Earlier work in educational sentiment analysis has moved from lexicon-based approaches toward machine learning and deep learning models (Dalipi et al., 2021). Models based on Bidirectional Encoder Representations from Transformers (BERT) have improved the representation of contextual semantics in massive open online course (MOOC) discussions and have been used to recognize emotional and cognitive engagement (Liu et al., 2022). Aspect-based and opinion-mining approaches further show that educational texts can contain evaluative cues related to specific learning experiences rather than only overall polarity (Chen et al., 2024; Koufakou, 2024).

In TPD-oriented online professional learning, text is especially relevant because participants often produce discourse through topic discussions, reflective summaries, learning reflections, and interactive comments. These texts are not designed as emotion-elicitation materials, but they are produced in activities that require participants to evaluate learning content, describe difficulties, express perceived gains, and connect course resources to teaching practice. Therefore, textual data can reveal affective-appraisal cues such as recognition, uncertainty, confidence, dissatisfaction, frustration, perceived usefulness, and professional gain. The interpretation remains bounded: such texts are treated as observable professional discourse, not as transparent records of private emotional experience.

Multimodal emotion recognition has sought to strengthen inference by combining text with speech, video, facial expression, physiological signals, and other modalities. Studies using classroom video, speech, and multimodal learning analytics demonstrate the potential of richer data for identifying affective and engagement-related states (M. Li et al., 2022; Sümer et al., 2021; Yan et al., 2024). However, multimodal systems also raise challenges of synchronization, governance, privacy, implementation cost, and ecological sustainability in authentic learning environments (Mohammadi et al., 2025; Prinsloo et al., 2023; Samuelsen et al., 2019; Schneider, 2024). These challenges are particularly important in large-scale professional learning platforms, where low-intrusion and routinely available data sources are often more feasible than sensor-based data collection.

Platform interaction logs provide such a source of process evidence. Clickstream and log data can capture resource access, task completion, session duration, interaction frequency, and changes in learning pace. In learning analytics, these traces have been used to understand self-regulated learning, time management, engagement, and persistence (Baker et al., 2020; Cao et al., 2023; Cristea et al., 2024; Q. Li et al., 2020). The psychological meaning of behavioral traces is inherently context-dependent, as similar activity patterns may reflect different learner states. When aligned with textual evidence and task context, logs can therefore provide complementary information about participation intensity, continuity, rhythm, and behavioral activation.

Taken together, the literature suggests that scalable affective-appraisal analytics in TPD-oriented online professional learning requires three forms of conceptual alignment. First, the representational framework must distinguish affective dimensions from appraisal-related value judgments. Second, the evidence base must treat texts and logs as indirect but meaningful traces of professional learning experience. Third, the modeling strategy must preserve temporal and contextual information rather than reducing all data to static polarity labels. These requirements motivate a four-dimensional affective-appraisal framework and a text-log fusion approach for week-level analysis.

## 3. Materials and Methods

**Figure 1:**
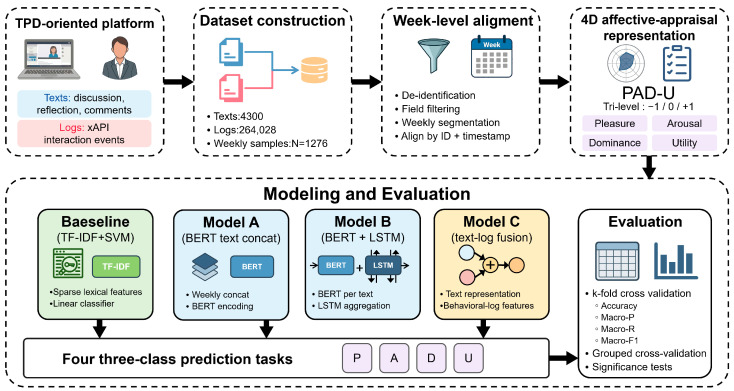
Overall workflow for constructing weekly affective-appraisal profiles from textual data and platform interaction logs in TPD-oriented online professional learning. Arrows indicate the direction of the analytical workflow. Abbreviations: TPD, teacher professional development; xAPI, Experience API; TF-IDF+SVM, term frequency–inverse document frequency plus support vector machine; BERT, Bidirectional Encoder Representations from Transformers; LSTM, long short-term memory.

Figure 1 presents the workflow for constructing and evaluating weekly affective-appraisal profiles from naturally generated platform data. The workflow begins with two data sources from a TPD-oriented online professional learning platform: textual artefacts and Experience API (xAPI)-based interaction logs. After dataset construction, de-identification, and weekly alignment by participant ID and timestamp, the data are represented within a four-dimensional affective-appraisal scheme comprising pleasure, arousal, dominance, and utility. Four modeling strategies are then compared under participant-level grouped cross-validation: a term frequency–inverse document frequency plus support vector machine (TF-IDF+SVM) baseline, a BERT-based weekly text concatenation model, a BERT–long short-term memory (LSTM) sequential text model, and a text-log fusion model. Each strategy is evaluated through four independent three-class prediction tasks corresponding to the four dimensions.

### 3.1. Dataset Construction

The empirical data were collected from a 21-week compulsory undergraduate course, Application of Modern Educational Technology, offered through a university platform to third-year educational technology majors. The course was designed for teacher education students and incorporated video-based learning, resource reading, topic discussions, reflective writing, and interaction within discussion communities. A total of 107 pre-service teachers aged 19 to 21 years participated in the study. Although they were not in-service teachers, they had accumulated initial teaching experience through school-based practicum. The empirical setting is therefore described as TPD-oriented professional learning rather than as an in-service TPD program. The course nevertheless provided a professional learning context characterized by sustained participation, reflection on teaching-related content, collaborative discussion, and practice-oriented evaluation.

Participation in the research was voluntary. All enrolled students provided informed consent before the course began, and no participant withdrew from the study during the 21-week period. Participants were informed that their textual outputs and platform interaction logs could be used for research on affective-appraisal analysis, that they could withdraw from the study without academic penalty, and that no further data would be collected from participants after withdrawal. The data used for analysis were de-identified before modeling, and a unified participant code was used as the sole identifier for each participant.

The textual data consisted of topic discussions, reflective summaries, learning reflections, and interactive comments produced during the course. In total, 4300 pieces of text were collected. The log dataset was exported from the platform backend in JavaScript Object Notation (JSON) format compliant with the Experience API (xAPI) specification and included 264,028 interaction records. The raw logs contained fields such as actor, timestamp, action type, and target object. The preprocessing stage retained the fields required for alignment and feature construction, including participant ID, timestamp, action type, and target object.

Textual data and interaction logs were segmented and aligned at the weekly level according to participant ID and timestamp. Weekly aggregation was used because the 21-week course was organized around weekly learning cycles, and each week involved video learning, resource reading, topic discussion, and/or reflective writing activities that generated both textual and behavioral evidence. The original participant-level sample included all 107 consenting pre-service teachers. At the teacher-week level, weeks without valid textual data or alignable interaction-log records were excluded because they could not support four-dimensional annotation or text-log fusion. After de-identification, cleaning, and week-level alignment, the final dataset contained 1276 valid teacher-week samples for model training and evaluation.

### 3.2. Four-Dimensional Affective-Appraisal Representation and Annotation

The label system was designed to support fine-grained but interpretable analysis of affective and appraisal-related cues in TPD-oriented professional learning texts. Building on the PAD framework (Mehrabian, 1996), pleasure, arousal, and dominance were used to represent affective valence, activation intensity, and perceived control. Utility was incorporated as an appraisal-related dimension that captures perceived usefulness, value judgment, and sense of professional gain. This dimension is theoretically aligned with value appraisal in educational emotion theory (Pekrun, 2006) and with perceived usefulness in technology acceptance research (Davis, 1989; Granić & Marangunić, 2019).

Each dimension was encoded independently using a three-level scheme, {−1, 0, 1}, corresponding to low or negative, neutral or non-salient, and high or positive states. The three-level scheme was adopted as an operational compromise between binary polarity classification and continuous scoring. It allows weak or non-salient cues to be represented explicitly while maintaining a level of granularity that is feasible for annotation and supervised classification. This coding strategy provides a tractable basis for modeling low-intensity and evaluative expressions in professional learning texts. Table 1 presents the four-dimensional affective-appraisal representation and the semantic interpretation of the three-level values. This framework was used to guide subsequent annotation and modeling in a consistent manner and to ensure that both the textual data and the multi-source data fusion model employed a unified labeling system.

The four dimensions can be combined to describe affective-appraisal profiles rather than deterministic equivalents of discrete emotions. The illustrative profiles in Table 2 are selected to represent common and theoretically interpretable configurations in professional learning texts, including neutral or non-salient expression, uniformly positive high-gain engagement, positive but low-arousal learning, challenging but useful learning, high activation with low perceived control, low-salience participation, and low-utility disengagement. The profile labels are descriptive interpretive labels based on the relative configuration of pleasure, arousal, dominance, and utility; they are not additional target classes, diagnostic categories, or exhaustive emotional states. They provide an interpretive bridge between the four independent dimensional labels and the later case-based examples.

Each text was annotated independently on the four dimensions. Values were assigned according to dimension–cue–value rules, with attention to affective words, evaluative statements, tone intensity, expressions of control or self-efficacy, and statements of usefulness or professional gain. When a text contained multiple evaluative targets or mixed expressions, annotation focused on the dominant evaluative target and the dominant affective-appraisal tendency. Texts with clear internal shifts were segmented into more coherent units during preprocessing when necessary. The annotation manual was organized around dimension–cue–value mappings, covering affective words, tone intensity, expressions of confidence or helplessness, and statements of usefulness, value, gain, or dissatisfaction.

Three trained annotators with disciplinary backgrounds in educational technology, learning analytics, and educational psychology independently coded the texts according to the four-dimensional scheme. Before formal annotation, the annotators received unified training and conducted pilot annotation using representative examples. The annotation manual was reviewed by two experts in educational psychology and learning analytics, who examined the clarity of the dimensional definitions, the appropriateness of cue-value mappings, and the treatment of mixed or ambiguous expressions. All revisions to the manual were completed through consensus before formal annotation. To assess inter-rater reliability, 30% of the texts were randomly selected and independently annotated by all three annotators. Fleiss’ kappa was calculated separately for the four dimensions (Fleiss, 1971), yielding 0.83 for pleasure, 0.75 for arousal, 0.76 for dominance, and 0.81 for utility. Disagreements were first discussed among the annotators; unresolved cases were adjudicated by an expert member of the research team. Final labels were determined through consensus-based adjudication. Table 3 summarizes the three-level decision criteria for the four-dimensional labels and served as the core basis for annotation.

The annotated label distribution before resampling is shown in Table 4. Class imbalance was observed across all four dimensions, with negative labels representing the smallest category in each case. Pleasure was characterized by a particularly large proportion of neutral or non-salient labels, whereas arousal, dominance, and utility contained higher proportions of positive or high-level labels. This pattern reflects the reflective and evaluative nature of professional learning texts, in which participants often expressed perceived gain, agency, or activation more explicitly than negative affect. The imbalance was addressed during model evaluation through training-fold-only undersampling.

### 3.3. Week-Level Affective-Appraisal Modeling with Text-Log Fusion

**Figure 2:**
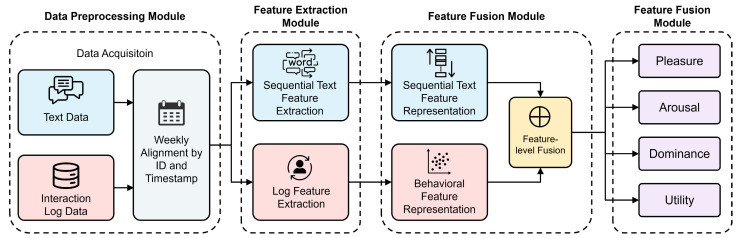
Architecture of the text-log fusion model, including weekly alignment, text and log feature extraction, feature-level fusion, and four-dimensional prediction.

Figure 2 illustrates the architecture of the text-log fusion model. The model operates at the teacher-week level. For each participant and week, the textual artefacts produced during that week and the corresponding interaction-log features were treated as one aligned sample. The text branch extracts contextual and sequential textual representations, whereas the log branch derives session-based behavioral features from platform interaction records. These representations are fused at the feature level and used to predict the three-class labels for pleasure, arousal, dominance, and utility.

Modeling was conducted at the weekly level, with the set of texts produced by a participant within a given week and the corresponding interaction-log features aggregated as a multi-source input for the same instance. Let the text set in week t be denoted as $X_{t}={\left\{x_{t,i}\right\}}_{i=1}^{n_{t}}$, and let the interaction-log feature vector be denoted as $B_{t}$. The corresponding four-dimensional affective-appraisal is defined as

$$y_{t}=\left(y_{t}^{v},y_{t}^{a},y_{t}^{d},y_{t}^{u}\right),y_{t}^{k}\in \left\{-1,0,1\right\},k\in \left\{v,a,d,u\right\}$$

where v,a,d and u represent pleasure, arousal, dominance, and utility, respectively. The objective of the model is to learn a mapping from $X_{t},B_{t}$ to $y_{t}$, and to output, for each of the four dimensions, the predicted probability distribution over the three-class space as well as the corresponding discrete prediction. To quantify the gains brought by interaction-log features, a text-only baseline model was also constructed. The architecture supports a sequential text-only variant and a text-log fusion variant, which share the same prediction space and differ in whether the interaction-log branch is included.

The text branch adopts a two-stage representation strategy in order to capture both the contextual semantic information of individual texts and the sequential information across multiple texts produced within the same week. For each text $x_{t,i}$, a pre-trained language model is used to obtain its contextualized vector representation. BERT-Base-Chinese was selected because all texts in the corpus were written in Chinese and consisted mainly of short reflective, evaluative, and discussion-based entries. The model provides contextual token representations suitable for capturing semantic and evaluative cues in Chinese educational discourse, while remaining computationally feasible for repeated grouped cross-validation. Although newer Chinese Transformer variants may offer stronger language representations, the aim of this study was to compare textual aggregation and text-log fusion strategies under a stable and widely used encoder rather than to optimize language-model architecture. A maximum token length of 512 is adopted to satisfy the model input constraints. The text representation is taken from the final hidden-state vector corresponding to the [CLS] token in the BERT output, resulting in a 768-dimensional embedding. Thus, for each text, the representation is defined as

$$e_{t,i}=BERT\left(x_{t,i}\right),e_{t,i}\in R^{768}$$

The sequence of text embeddings within the same week was then arranged chronologically and passed to an LSTM (Long Short-Term Memory) network to obtain a week-level textual representation:

$$z_{t}^{text}=LSTM\left(e_{t,1},e_{t,2},\ldots ,e_{t,n_{t}}\right)$$

The maximum number of texts per week was set to five based on the empirical distribution of weekly text counts and computational considerations. Of the 1276 teacher-week samples, 1136 samples (89.0%) contained no more than five texts, and only 140 samples (11.0%) exceeded this threshold. At this setting, 4092 of the 4300 textual records were retained, corresponding to a retained-text ratio of 95.2%. The threshold therefore preserved most weekly textual evidence while preventing a small number of highly active weeks from disproportionately increasing sequence length and computational cost. When a participant produced more than five texts in a week, the chronological sequence was truncated to the maximum length. Sensitivity analyses were also conducted using alternative maximum sequence lengths of 3, 7, and 10 texts to examine whether the model performance was sensitive to this setting.

The log branch transforms platform interaction records into an interpretable weekly behavioral feature vector. For each participant-week, interaction events were sorted by timestamp, and the time intervals between consecutive events were used to segment activity into learning sessions. The 3 h threshold was used as the default setting because it provided a practical boundary between continuous learning activity and separate learning sessions in the platform context, and its suitability was further examined through robustness analyses using 1 h to 4 h thresholds. Five weekly features were constructed: number of actions, number of sessions, total duration, average session duration, and standard deviation of session duration. These features were selected because they provide interpretable indicators of participation frequency, session structure, duration, and temporal regularity, while avoiding a high-dimensional behavioral feature space given the moderate participant-level sample size. These features characterize interaction intensity, continuity, and temporal stability, forming the weekly log-based feature vector $z_{t}^{log}\in R^{5}$.

To eliminate differences in scale across features and improve the model’s efficiency in learning feature relationships, the behavioral features were standardized. Let a behavioral feature be denoted as x, with mean μ and standard σ; its standardized form is computed as $\tilde{x}=\frac{x-\mu }{\sigma }$. The standardized behavioral vector was then used as the weekly behavioral representation and fed into the fusion module.

At the fusion stage, the weekly text representation and the standardized log representation are concatenated along the feature dimension to form a fused week-level representation $z_{t}=\left[z_{t}^{text};z_{t}^{log}\right]$. Feature-level concatenation combines semantic evidence from texts with process evidence from platform activity after weekly alignment. The sequential text-only variant uses $z_{t}^{text}$ as input, whereas the fusion variant uses the concatenated representation for the same four three-class prediction tasks.

For prediction, four independent three-class SVM classifiers were trained, one for each affective-appraisal dimension. This design preserves the dimensional structure of the representation and allows pleasure, arousal, dominance, and utility to be evaluated separately. SVM was selected because the study involved a moderate number of teacher-week samples and high-dimensional representations derived from BERT, LSTM aggregation, and behavioral-log features. In this setting, SVM provides a stable discriminative classifier with relatively low hyperparameter complexity and a lower risk of overfitting than a fully fine-tuned neural classifier. The four classifiers share the same label space {−1, 0, 1}, corresponding to low or negative, neutral or non-salient, and high or positive labels.

Implementation details were specified to support reproducibility. The experiments used Python 3.8, PyTorch 2.0.1, Transformers, and scikit-learn on Windows 11 with an Intel i7-12700F CPU and an NVIDIA RTX 3060 Ti GPU. BERT-Base-Chinese was used as the text encoder with max_length = 512. The LSTM hidden size was set to 768, the default maximum weekly text sequence length was 5, the optimizer was AdamW, and the random seed was fixed across repeated runs. Evaluation adopted 5 repeats × 5-fold participant-level grouped cross-validation, with participant ID used as the grouping variable to prevent teacher-week samples from the same participant appearing in both training and test folds. Within each training fold, random undersampling was applied to the majority classes for each dimension so that the class sizes were matched to the minority class in the corresponding training subset. This procedure was repeated separately for each cross-validation split using a fixed random seed. The validation and test folds were left unchanged to preserve the natural class distribution during evaluation. This strategy reduced class imbalance during model training while avoiding information leakage from the evaluation data.

## 4. Results

Model performance was evaluated using participant-level grouped cross-validation. In addition to the three main modeling strategies, a TF-IDF+SVM classifier was included as a conventional external baseline based on sparse lexical features. Model A denotes the BERT-based weekly text concatenation model and serves as the non-sequential text baseline. Model B denotes the BERT-LSTM sequential text model, which represents each text with BERT and aggregates weekly textual sequences using LSTM. Model C denotes the text-log fusion model, which extends Model B by incorporating interaction-log features through feature-level fusion. Performance was assessed using macro-averaged precision (Macro-P), macro-averaged recall (Macro-R), macro-averaged F1 score (Macro-F1), and overall accuracy (Accuracy) for each affective-appraisal dimension.

The results are organized around the three analytical aims of the study. The operationalization of the four-dimensional affective-appraisal representation is supported by the annotation criteria, label distributions, and reliability evidence reported in Section 3.2. The contribution of intra-week sequential text aggregation is evaluated through the comparison between Model A and Model B, whereas the additional contribution of interaction logs is evaluated through the comparison between Model B and Model C, together with robustness, sensitivity, and confusion-matrix analyses. The representative cases at the end of this section illustrate how the dimensional outputs can be interpreted as affective-appraisal profiles.

Table 5 summarizes the main performance results. The baseline provided a conventional reference point for sparse-feature text classification, but it was generally weaker than the contextual and sequential models, especially in Macro-F1. Model A did not consistently improve over the baseline. Its pleasure Macro-F1 was lower than that of the baseline, and its utility Accuracy was also slightly lower. This suggests that directly concatenating all texts produced within a week does not necessarily preserve the affective structure of weekly discourse. For teacher-week samples containing multiple short reflections, discussions, and comments, direct concatenation may obscure the order and relative contribution of individual textual artefacts, while also being constrained by the maximum input length of the language model.

The comparison between Model A and Model B indicates that intra-week sequential aggregation contributed substantially to recognition performance. Model B improved Macro-F1 across all four dimensions, with gains of 0.1798 for pleasure, 0.1702 for arousal, 0.0924 for dominance, and 0.0899 for utility. The largest gains occurred for pleasure and arousal, suggesting that affective valence and activation were particularly sensitive to the temporal organization of weekly textual production. Accuracy also increased across all dimensions, indicating that the sequential model did not merely improve minority-class sensitivity but also enhanced overall prediction at the teacher-week level.

Model C produced the strongest overall performance. It achieved the highest Macro-F1 and Accuracy for all four dimensions and the highest Macro-P across the four dimensions. The only exception to a uniform advantage across all metrics was Macro-R for arousal, where Model B slightly exceeded Model C. This pattern suggests that log-based features improved overall precision and sample-level correctness, while the recall of arousal remained more sensitive to category boundaries. The largest Accuracy gains from Model B to Model C were observed for arousal and utility, both increasing by 0.0508. This is consistent with the role of interaction logs as process evidence: arousal is partly reflected in behavioral activation and learning rhythm, whereas utility may be associated with sustained access, repeated participation, and continued engagement with professional learning resources.

**Figure 3:**
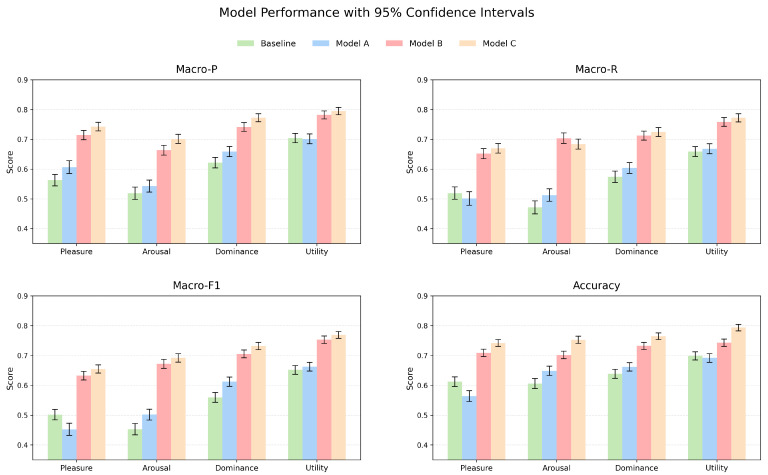
Macro-P, Macro-R, Macro-F1, and Accuracy with 95% confidence intervals across repeated grouped cross-validation estimates.

Figure 3 provides a visual summary of model performance with 95% confidence intervals across repeated grouped cross-validation estimates. The confidence intervals show a stable ordering of the models for most metrics and dimensions. Model B and Model C generally exhibited higher and less dispersed estimates than the baseline and Model A, indicating that sequential aggregation and text-log fusion improved not only mean performance but also cross-validation stability. The intervals for Model B and Model C overlap in several cases, particularly for Macro-F1, which is consistent with the smaller incremental gains obtained after adding interaction logs. In contrast, the separation between Model A and Model B is more visible, especially for pleasure and arousal. Full mean ± standard deviation (SD) values and confidence intervals are reported in Appendix A.

Statistical comparisons were conducted on repeated grouped cross-validation results with Holm–Bonferroni correction. The analysis focused on two comparisons aligned with the modeling sequence: Model B versus Model A, which estimates the contribution of intra-week sequential aggregation, and Model C versus Model B, which estimates the additional contribution of interaction-log features. The complete pairwise results are reported in Appendix A.

The statistical pattern supports the contribution of sequential text modeling. Model B significantly outperformed Model A in Macro-F1 across all four dimensions, with adjusted *p*-values ranging from 0.002 to 0.027. The gains were most pronounced for pleasure and arousal, suggesting that sequential aggregation was especially useful when affective cues were distributed across multiple weekly textual artefacts rather than concentrated in a single entry. This pattern is consistent with the descriptive results in Table 5, where Model B produced large Macro-F1 increases over Model A for pleasure and arousal and smaller but consistent gains for dominance and utility.

The contribution of interaction logs showed a different performance profile. Model C produced significant Accuracy gains over Model B across all four dimensions, whereas Macro-F1 improvements were smaller and reached statistical significance only for dominance. This distinction indicates that log features primarily strengthened overall teacher-week classification consistency rather than uniformly improving class-balanced recognition. In substantive terms, interaction logs provided information about participation intensity, session continuity, and behavioral activation, which improved overall classification stability. However, distinguishing less frequent categories still depended largely on whether the texts contained explicit affective or appraisal-related cues.

Robustness was examined by varying the session-segmentation threshold from 1 to 4 h while keeping the remaining modeling settings unchanged. Table 6 reports Macro-F1 and Accuracy for Model C under each threshold; the full robustness results, including Macro-P and Macro-R, are provided in Table A5. This analysis focuses on whether the effect of interaction-log features depends on a specific inactivity threshold for defining learning sessions.

The results remained stable across the four thresholds. The 3-h threshold yielded the best Macro-F1 and Accuracy for all four dimensions, but the differences across thresholds were modest. For example, Macro-F1 varied within a narrow range for pleasure (0.6462–0.6551), arousal (0.6828–0.6917), dominance (0.7224–0.7317), and utility (0.7589–0.7689). Accuracy showed a similarly stable pattern. These findings indicate that the contribution of interaction-log features was not driven by a single arbitrary segmentation setting. Instead, the log branch captured relatively stable process indicators across plausible definitions of learning sessions.

Additional sensitivity analyses examined whether the maximum weekly text sequence length affected model performance. Table 7 compares maximum sequence lengths of 3, 5, 7, and 10 texts; length 10 was equivalent to no truncation in this dataset because no teacher-week sample contained more than ten texts.

The sensitivity results remained stable across sequence-length settings. Reducing the maximum length to three texts produced visible decreases in Macro-F1 and Accuracy across all four dimensions. By contrast, increasing the maximum length from five to seven or ten produced only negligible changes, with differences of less than 0.001 in both Macro-F1 and Accuracy. Because the length-five setting retained 95.2% of all texts while yielding performance comparable to longer sequence settings, it was used as the maximum sequence length in the main analyses.

**Figure 4:**
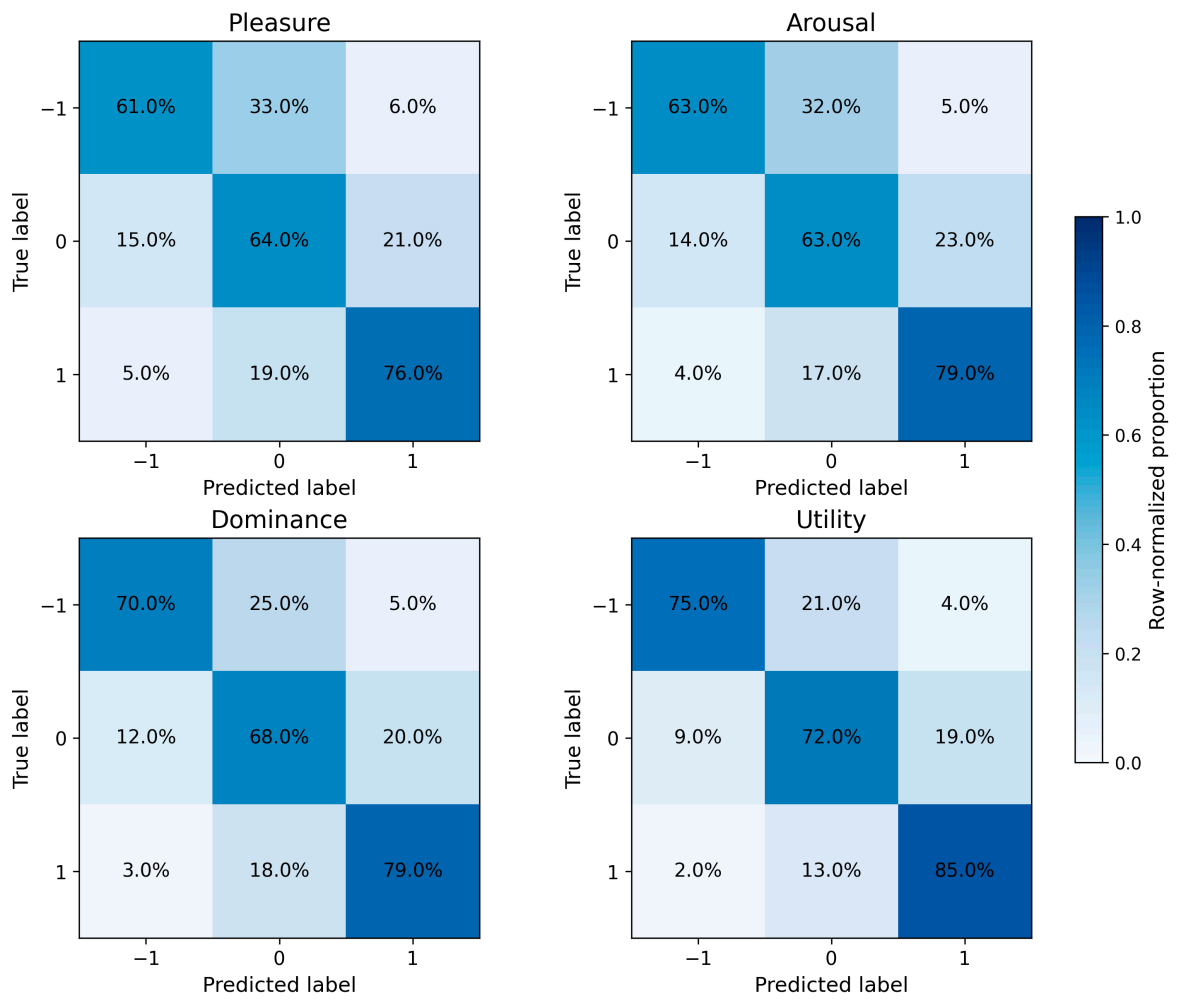
Row-normalized confusion-matrix proportions for Model C across the four affective-appraisal dimensions.

Figure 4 presents row-normalized confusion matrices for Model C. Correct classifications were concentrated along the diagonal across the four dimensions, indicating that the fusion model captured the dominant label structure in the teacher-week samples. Errors occurred mainly between adjacent categories rather than between the two extremes. This pattern is consistent with the nature of affective-appraisal annotation, where weak, mixed, or restrained expressions may fall near category boundaries. It also suggests that the model rarely confused clearly low or negative profiles with clearly high or positive profiles.

The confusion matrices also reveal dimension-specific patterns. Utility showed the clearest diagonal concentration, particularly for the positive category, indicating that perceived usefulness and professional gain were comparatively identifiable when textual evidence was combined with interaction-log features. Dominance also showed a relatively clear pattern, although neutral cases were sometimes confused with positive cases, reflecting the difficulty of distinguishing non-salient control cues from mild expressions of agency. Arousal displayed more adjacent-category confusion, especially between neutral and high activation, which is consistent with the multidirectional meaning of activation: high arousal may reflect enthusiasm, effort, tension, or uncertainty depending on the surrounding textual and behavioral context. Pleasure showed a similar boundary issue around neutral expressions, reflecting the prevalence of reflective and evaluative texts with limited overt affective wording.

Table 8 presents representative teacher-week profiles to illustrate how empirically observed model outputs relate to the profile logic introduced in Table 2. The cases were not selected to validate an exhaustive profile taxonomy; rather, they show how the four-dimensional coordinates can be interpreted in relation to differentiated patterns such as positive high-gain engagement, challenging but useful learning, and weak textual expression accompanied by behavioral activation. These examples are presented as interpretive illustrations of the model output rather than as independent validation evidence.

The cases show how the four-dimensional profiles distinguish patterns that would be obscured by coarse positive–negative classification and how the empirical examples relate to the illustrative profiles in Table 2. Case 1 corresponds to the positive high-gain engagement profile: the textual evidence expressed positive reflection and perceived learning benefits, while the log pattern showed repeated access to course materials and continued activity. Case 2 aligns with the challenging but useful learning profile, in which difficulty and uncertainty coexisted with perceived usefulness and sustained participation. This profile is particularly informative because low pleasure and low dominance were not accompanied by low utility; instead, the participant appeared to experience the activity as challenging while still recognizing its professional value. Case 3 further illustrates the flexibility of the profile-based representation. Although the textual expression was relatively restrained, frequent actions and repeated sessions indicated behavioral activation and continued engagement, while the utility dimension suggested perceived usefulness or learning value. This pattern extends the illustrative configurations in Table 2 by showing how naturally occurring teacher-week profiles may combine weak textual affect, sustained behavioral participation, and positive value appraisal without assuming a simple one-to-one alignment among textual cues, behavioral traces, and value appraisal.

Taken together, the results indicate that the main performance gain from Model B was associated with preserving the temporal structure of weekly textual production, whereas the additional contribution of Model C was associated with integrating process evidence from platform interaction logs. These results directly address the second and third objectives concerning sequential text aggregation and text-log fusion. The profile-level interpretation of the outputs addresses the first objective by showing how affective valence, activation, perceived control, and perceived usefulness can diverge in professional learning. Rather than treating the profiles as validated emotional states, the results support their use as descriptive affective-appraisal configurations for interpreting the mixed and evaluative character of pre-service teachers’ online professional learning experiences.

## 5. Discussion

The findings indicate that fine-grained affective-appraisal analytics in TPD-oriented online professional learning requires conceptual alignment between the representational framework, the temporal structure of textual evidence, and process traces from platform interaction logs. Professional learning texts are not equivalent to direct emotional reports; they are reflective, evaluative, and shaped by professional norms (Ehlert et al., 2025; Nash, 2022). Within this communicative context, the four-dimensional framework provided a way to model affective valence, activation, perceived control, and value appraisal as related but distinguishable aspects of professional learning experience. In this sense, the framework contributes to the assessment of teacher emotions by modeling observable affective-appraisal configurations in professional learning discourse, while avoiding claims about direct measurement of latent emotional states.

A central implication concerns the role of utility in the affective-appraisal framework. The results and representative cases show that perceived usefulness and professional gain may diverge from pleasure or perceived control. Participants could describe a learning activity as difficult or uncertain while still treating it as valuable for professional growth. This pattern supports the conceptualization of utility as an appraisal-related dimension that complements pleasure, arousal, and dominance rather than as a simple indicator of positive affect. It also aligns with educational emotion theory, in which value appraisal shapes emotional experience, and with technology acceptance research, in which perceived usefulness is central to engagement with digital learning environments (Pekrun, 2006; Davis, 1989; Granić & Marangunić, 2019).

The performance pattern also highlights the importance of preserving the intra-week structure of textual production. Direct weekly text concatenation did not consistently outperform the conventional sparse-feature baseline, suggesting that simply merging all texts in a week may obscure the local organization of reflections, discussions, and comments. In contrast, sequential aggregation produced consistent gains, particularly for pleasure and arousal. This indicates that affective-appraisal cues in professional learning are often distributed across multiple short textual artefacts, and that their order and accumulation across a weekly learning cycle carry information that can be lost in a single merged representation.

The integration of interaction logs further clarifies how process evidence can complement textual evidence. The fusion model improved overall classification stability, especially in accuracy, and the gains were most visible for arousal and utility. These dimensions are closely connected to behavioral activation, participation rhythm, and sustained engagement, which are only partially observable in written discourse. Interaction logs therefore extend the evidence base by capturing patterns of access, session continuity, and temporal stability. At the same time, the smaller gains in class-balanced performance indicate that log data do not replace textual interpretation; distinctions involving less frequent or weakly expressed categories still depend on the specificity of affective and appraisal-related cues in the texts.

The error patterns provide a further basis for interpreting the model outputs. The row-normalized confusion matrices showed that most errors occurred between adjacent categories rather than between low and high extremes. This pattern is consistent with the nature of affective-appraisal annotation, where restrained professional discourse and mixed evaluative statements may lie close to category boundaries. Utility showed the clearest diagonal pattern, suggesting that value appraisal and perceived gain were relatively identifiable when textual and behavioral evidence were combined. Arousal and pleasure involved more adjacent-category confusion, reflecting the fact that activation and affective tone can be expressed indirectly and may vary with task context, perceived challenge, and communicative style.

These findings have practical implications for emotion-aware analytics in online professional learning environments. A weekly affective-appraisal profile can provide descriptive information about patterns such as low perceived usefulness, reduced control, high activation, or positive high-gain engagement. The relevance of these outputs for assessing teacher emotions lies in their capacity to characterize observable affective-appraisal configurations in professional learning discourse, rather than to diagnose latent emotional states. Such information may help instructors and course organizers identify when reflective scaffolding, task clarification, resource adjustment, or timely support is needed. The use of naturally generated texts and routine platform logs also makes the approach comparatively low-intrusion and scalable. Nevertheless, the outputs should be interpreted as decision-support indicators rather than direct measurements of private emotional states or as grounds for automated evaluation of individual participants. This caution is also relevant to the precision–recall pattern observed in the results. Although interaction logs improved overall accuracy and precision, recall did not improve uniformly across all dimensions. In practical use, a model with lower recall may fail to flag some low-salience or at-risk affective-appraisal profiles, whereas an overly sensitive model may generate unnecessary follow-up and increase perceptions of monitoring. Model outputs should therefore be treated as preliminary signals for low-stakes, human-mediated support rather than as automatic screening results.

Several limitations delimit the interpretation of the findings. The empirical setting involved pre-service teachers in a single TPD-oriented course. Although the participants had accumulated initial teaching experience through school-based practicum, the setting remains distinct from in-service professional development. The week-level unit of analysis also smooths over shorter affective fluctuations that may occur within specific tasks or discussions. In addition, the log branch relied on a compact set of handcrafted session-based features, and the annotation scheme required expert judgment despite satisfactory inter-rater reliability. The profile-based operationalization also remains a pragmatic analytic representation rather than a comprehensive theory of teacher emotion. The study did not include participant self-reports, experience-sampling ratings, or external criterion measures that could be directly compared with the four-dimensional affective-appraisal labels. The validity evidence should therefore be understood as internal evidence derived from theory-driven coding, expert consultation, inter-rater reliability, model performance, and illustrative profile analysis, rather than as criterion validation against participants’ self-reported emotional experiences. Future research should examine the framework in diverse in-service TPD contexts, compare additional temporal and multimodal modeling strategies, incorporate participant ratings or experience-sampling measures, and investigate how affective-appraisal profiles can inform ethically responsible forms of adaptive support.

## 6. Conclusions

This study developed and evaluated a four-dimensional affective-appraisal framework for analyzing emotion-related cues in TPD-oriented online professional learning. By integrating utility with pleasure, arousal, and dominance, the framework captures affective and appraisal-related cues in professional learning discourse and supports profile-based interpretation of mixed evaluative patterns. Using data from an authentic online course for pre-service teachers, the results showed that intra-week sequential modeling improved recognition performance over conventional and non-sequential text-based approaches, indicating the importance of preserving the temporal organization of weekly textual production.

The integration of platform interaction logs provided complementary process evidence and improved overall classification stability, particularly for arousal and utility. These findings suggest that text-log fusion can support scalable, low-intrusion, and context-sensitive affective-appraisal analytics in pre-service teacher professional learning. The framework offers an interpretable basis for monitoring professional learning processes and informing support design, while further validation in broader in-service TPD settings is needed before it can be generalized to wider teacher professional development contexts.

## Figures and Tables

**Table 1:** Four-dimensional affective-appraisal representation and three-level coding scheme.

Dimension	Meaning in the TPD-Oriented Professional Learning	−1	0	1
Pleasure	Overall affective valence toward the professional learning experience	Negative	Neutral/not salient	Positive
Arousal	Activation intensity and strength of affective or evaluative expression	Low arousal	Moderate/not salient	High arousal
Dominance	Perceived control, agency, confidence, or helplessness in the learning process	Constrained/helpless	Not salient	In control/confident
Utility	Perceived usefulness, value judgment, and sense of professional gain	Low value/perceived uselessness	Not salient	Sense of gain/recognition

**Table 2:** Illustrative profile labels and coordinate patterns within the four-dimensional affective-appraisal representation.

Illustrative Profile	Pleasure	Arousal	Dominance	Utility
Neutral/non-salient profile	0	0	0	0
Positive high-gain engagement	1	1	1	1
Focused low-arousal learning	1	0	1	1
Challenging but useful learning	−1	1	−1	1
High-arousal uncertainty	0	1	−1	0
Low-salience participation	0	−1	0	0
Low-utility disengaged profile	−1	−1	−1	−1

**Table 3:** Four-dimensional annotation guidelines for teacher professional development texts.

Dimension	Label	Annotation Criteria and Cues
Pleasure	−1	Negative affective words, negative evaluations, dissatisfaction, or an overall negative orientation
	0	No salient positive or negative cue, or balanced affective orientation
	1	Positive affective words, affirmative evaluations, optimism, satisfaction, or an overall positive orientation
Arousal	−1	Low energy, restrained tone, fatigue, passivity, or weak activation
	0	No salient intensity cue, or moderate activation
	1	Strong tone, urgency, excitement, tension, repeated emphasis, or high activation
Dominance	−1	Helplessness, lack of control, uncertainty, dependence, or perceived constraint
	0	No salient cue of control or helplessness
	1	Confidence, agency, initiative, self-efficacy, or perceived control
Utility	−1	Low value, perceived uselessness, lack of gain, or dissatisfaction with usefulness
	0	No salient value judgment or perceived gain
	1	Perceived usefulness, professional gain, recognition, achievement, or transfer value

**Table 4:** Label distribution before resampling.

Dimension	−1	0	1	Total
Pleasure	161 (12.6%)	800 (62.7%)	315 (24.7%)	1276
Arousal	108 (8.5%)	506 (39.7%)	662 (51.9%)	1276
Dominance	166 (13.0%)	387 (30.3%)	723 (56.7%)	1276
Utility	158 (12.4%)	500 (39.2%)	618 (48.4%)	1276

**Table 5:** Macro-P, Macro-R, Macro-F1, and Accuracy across the four affective-appraisal dimensions.

Dimension	Model	Macro-P	Macro-R	Macro-F1	Accuracy
Pleasure	Baseline	0.5628	0.5194	0.5017	0.6125
	A	0.6063	0.5012	0.4526	0.5638
	B	0.7139	0.6518	0.6324	0.7087
	C	0.7426	0.6697	0.6551	0.7418
Arousal	Baseline	0.5186	0.4713	0.4528	0.6062
	A	0.5427	0.5128	0.5019	0.6489
	B	0.6635	0.7036	0.6721	0.7016
	C	0.7014	0.6839	0.6917	0.7524
Dominance	Baseline	0.6215	0.5742	0.5596	0.6387
	A	0.6589	0.6037	0.6124	0.6621
	B	0.7416	0.7125	0.7048	0.7319
	C	0.7723	0.7246	0.7317	0.7648
Utility	Baseline	0.7042	0.6587	0.6519	0.6984
	A	0.7015	0.6683	0.6627	0.6916
	B	0.7818	0.7584	0.7526	0.7427
	C	0.7946	0.7721	0.7689	0.7935

**Table 6:** Macro-F1 and Accuracy of Model C under different session-segmentation thresholds.

Dimension	Macro-F1				Accuracy			
	1 h	2 h	3 h	4 h	1 h	2 h	3 h	4 h
Pleasure	0.6462	0.6527	0.6551	0.6504	0.7284	0.7369	0.7418	0.7336
Arousal	0.6828	0.6889	0.6917	0.6865	0.7396	0.7481	0.7524	0.7443
Dominance	0.7224	0.7287	0.7317	0.7266	0.7516	0.7592	0.7648	0.7575
Utility	0.7589	0.7647	0.7689	0.7631	0.7807	0.7891	0.7935	0.7862

**Table 7:** Macro-F1 and Accuracy of Model C under different maximum weekly text sequence lengths.

Dimension	Macro-F1				Accuracy			
	Length = 3	Length = 5	Length = 7	Length = 10	Length = 3	Length = 5	Length = 7	Length = 10
Pleasure	0.6428	0.6551	0.6549	0.6544	0.7289	0.7418	0.7415	0.7410
Arousal	0.6795	0.6917	0.6920	0.6912	0.7398	0.7524	0.7528	0.7521
Dominance	0.7198	0.7317	0.7314	0.7310	0.7526	0.7648	0.7645	0.7641
Utility	0.7564	0.7689	0.7691	0.7685	0.7810	0.7935	0.7937	0.7932

**Table 8:** Representative teacher-week profiles and their relation to illustrative affective-appraisal patterns.

Case	Evidence and Interpretation	Four-Dimensional Profile			
		P	A	D	U
Case 1 (A000027, W1)	The texts expressed positive reflection and gains, while the logs showed repeated material access and sustained participation. The profile indicates high-gain engagement with strong perceived control.	1	1	1	1
Case 2 (A000056, W4)	The texts reflected difficulty and uncertainty but also perceived usefulness. Continued revisits in the logs indicate sustained participation despite low pleasure and low dominance.	−1	1	−1	1
Case 3 (A000073, W6)	The texts contained weak or restrained affective cues, whereas frequent actions and repeated sessions indicated behavioral activation and perceived usefulness.	0	1	0	1

## Data Availability

The data presented in this study are not publicly available due to privacy and ethical restrictions. Because the participants were students, the dataset includes student-generated learning texts and platform interaction records, and public release may compromise participant confidentiality and anonymity.

## Abbreviations

The following abbreviations are used in this manuscript:

TPD	Teacher Professional Development
PAD	Pleasure–Arousal–Dominance
TAM	Technology Acceptance Model
AEQ	Achievement Emotions Questionnaire
BERT	Bidirectional Encoder Representations from Transformers
LSTM	Long Short-Term Memory
SVM	Support Vector Machine
xAPI	Experience API
JSON	JavaScript Object Notation
Macro-P	Macro-Averaged Precision
Macro-R	Macro-Averaged Recall
Macro-F1	Macro-Averaged F1 Score

## Appendix A. Supplementary Model Evaluation

This appendix provides supplementary statistical information corresponding to the model comparisons reported in the Section 4. Table A1 and Table A2 report standard deviations and 95% confidence intervals. Table A3 and Table A4 provide the complete statistical comparisons, and Table A5 reports the full robustness results under different session-segmentation thresholds.

**Table A1:** Macro-F1 and Accuracy with standard deviations and 95% confidence intervals.

Dimension	Model	Macro-F1 Mean ± SD	Macro-F1 95% CI	Accuracy Mean ± SD	Accuracy 95% CI
Pleasure	Baseline	0.5017 ± 0.0430	[0.4839, 0.5195]	0.6125 ± 0.0390	[0.5964, 0.6286]
	A	0.4526 ± 0.0500	[0.4320, 0.4732]	0.5638 ± 0.0440	[0.5456, 0.5820]
	B	0.6324 ± 0.0350	[0.6180, 0.6468]	0.7087 ± 0.0300	[0.6963, 0.7211]
	C	0.6551 ± 0.0330	[0.6415, 0.6687]	0.7418 ± 0.0280	[0.7302, 0.7534]
Arousal	Baseline	0.4528 ± 0.0460	[0.4338, 0.4718]	0.6062 ± 0.0410	[0.5893, 0.6231]
	A	0.5019 ± 0.0440	[0.4837, 0.5201]	0.6489 ± 0.0380	[0.6332, 0.6646]
	B	0.6721 ± 0.0360	[0.6572, 0.6870]	0.7016 ± 0.0310	[0.6888, 0.7144]
	C	0.6917 ± 0.0340	[0.6777, 0.7057]	0.7524 ± 0.0290	[0.7404, 0.7644]
Dominance	Baseline	0.5596 ± 0.0400	[0.5431, 0.5761]	0.6387 ± 0.0360	[0.6238, 0.6536]
	A	0.6124 ± 0.0380	[0.5967, 0.6281]	0.6621 ± 0.0340	[0.6481, 0.6761]
	B	0.7048 ± 0.0320	[0.6916, 0.7180]	0.7319 ± 0.0290	[0.7199, 0.7439]
	C	0.7317 ± 0.0300	[0.7193, 0.7441]	0.7648 ± 0.0270	[0.7537, 0.7759]
Utility	Baseline	0.6519 ± 0.0360	[0.6370, 0.6668]	0.6984 ± 0.0330	[0.6848, 0.7120]
	A	0.6627 ± 0.0350	[0.6483, 0.6771]	0.6916 ± 0.0340	[0.6776, 0.7056]
	B	0.7526 ± 0.0300	[0.7402, 0.7650]	0.7427 ± 0.0300	[0.7303, 0.7551]
	C	0.7689 ± 0.0280	[0.7573, 0.7805]	0.7935 ± 0.0260	[0.7828, 0.8042]

**Table A2:** Macro-P and Macro-R with standard deviations and 95% confidence intervals.

Dimension	Model	Macro-P Mean ± SD	Macro-P 95% CI	Macro-R Mean ± SD	Macro-R 95% CI
Pleasure	Baseline	0.5628 ± 0.0470	[0.5434, 0.5822]	0.5194 ± 0.0500	[0.4988, 0.5400]
	A	0.6063 ± 0.0520	[0.5848, 0.6278]	0.5012 ± 0.0550	[0.4785, 0.5239]
	B	0.7139 ± 0.0380	[0.6982, 0.7296]	0.6518 ± 0.0410	[0.6349, 0.6687]
	C	0.7426 ± 0.0350	[0.7282, 0.7570]	0.6697 ± 0.0390	[0.6536, 0.6858]
Arousal	Baseline	0.5186 ± 0.0500	[0.4980, 0.5392]	0.4713 ± 0.0530	[0.4494, 0.4932]
	A	0.5427 ± 0.0490	[0.5225, 0.5629]	0.5128 ± 0.0510	[0.4917, 0.5339]
	B	0.6635 ± 0.0400	[0.6470, 0.6800]	0.7036 ± 0.0430	[0.6858, 0.7214]
	C	0.7014 ± 0.0370	[0.6861, 0.7167]	0.6839 ± 0.0410	[0.6670, 0.7008]
Dominance	Baseline	0.6215 ± 0.0430	[0.6037, 0.6393]	0.5742 ± 0.0460	[0.5552, 0.5932]
	A	0.6589 ± 0.0410	[0.6420, 0.6758]	0.6037 ± 0.0440	[0.5855, 0.6219]
	B	0.7416 ± 0.0350	[0.7272, 0.7560]	0.7125 ± 0.0370	[0.6972, 0.7278]
	C	0.7723 ± 0.0320	[0.7591, 0.7855]	0.7246 ± 0.0360	[0.7097, 0.7395]
Utility	Baseline	0.7042 ± 0.0380	[0.6885, 0.7199]	0.6587 ± 0.0400	[0.6422, 0.6752]
	A	0.7015 ± 0.0400	[0.6850, 0.7180]	0.6683 ± 0.0410	[0.6514, 0.6852]
	B	0.7818 ± 0.0330	[0.7682, 0.7954]	0.7584 ± 0.0350	[0.7440, 0.7728]
	C	0.7946 ± 0.0300	[0.7822, 0.8070]	0.7721 ± 0.0330	[0.7585, 0.7857]

**Table A3:** Significance tests for Macro-F1.

Dimension	Comparison	ΔMacro-F1	*p*-Value	Adjusted *p*-Value	Result
Pleasure	B − Baseline	+0.1307	0.001	0.004	Significant
Pleasure	B–A	+0.1798	<0.001	0.002	Significant
Pleasure	C–B	+0.0227	0.041	0.098	Not significant after correction
Arousal	B − Baseline	+0.2193	<0.001	<0.001	Significant
Arousal	B–A	+0.1702	<0.001	0.002	Significant
Arousal	C–B	+0.0196	0.033	0.083	Marginal/not significant after correction
Dominance	B − Baseline	+0.1452	0.001	0.004	Significant
Dominance	B–A	+0.0924	0.004	0.016	Significant
Dominance	C–B	+0.0269	0.014	0.048	Significant
Utility	B − Baseline	+0.1007	0.006	0.021	Significant
Utility	B–A	+0.0899	0.009	0.027	Significant
Utility	C–B	+0.0163	0.052	0.113	Not significant after correction

**Table A4:** Significance tests for Accuracy comparing Model C with Model B.

Dimension	Comparison	ΔAccuracy	*p*-Value	Adjusted *p*-Value	Interpretation
Pleasure	C–B	+0.0331	0.009	0.018	Significant
Arousal	C–B	+0.0508	0.002	0.008	Significant
Dominance	C–B	+0.0329	0.011	0.022	Significant
Utility	C–B	+0.0508	0.001	0.004	Significant

**Table A5:** Robustness check for session-segmentation thresholds in Model C.

Dimension	Threshold	Macro-P	Macro-R	Macro-F1	Accuracy
Pleasure	1 h	0.7318	0.6605	0.6462	0.7284
	2 h	0.7395	0.6668	0.6527	0.7369
	3 h	0.7426	0.6697	0.6551	0.7418
	4 h	0.7369	0.6641	0.6504	0.7336
Arousal	1 h	0.6907	0.6762	0.6828	0.7396
	2 h	0.6978	0.6814	0.6889	0.7481
	3 h	0.7014	0.6839	0.6917	0.7524
	4 h	0.6956	0.6798	0.6865	0.7443
Dominance	1 h	0.7609	0.7163	0.7224	0.7516
	2 h	0.7684	0.7215	0.7287	0.7592
	3 h	0.7723	0.7246	0.7317	0.7648
	4 h	0.7661	0.7198	0.7266	0.7575
Utility	1 h	0.7835	0.7624	0.7589	0.7807
	2 h	0.7902	0.7689	0.7647	0.7891
	3 h	0.7946	0.7721	0.7689	0.7935
	4 h	0.7887	0.7665	0.7631	0.7862

## References

[B1-behavsci-16-01025] Baker R., Xu D., Park J., Yu R., Li Q., Cung B., Fischer C., Rodriguez F., Warschauer M., Smyth P. (2020). The benefits and caveats of using clickstream data to understand student self-regulatory behaviors: Opening the black box of learning processes. International Journal of Educational Technology in Higher Education.

[B2-behavsci-16-01025] Cao T., Zhang Z., Chen W., Shu J. (2023). Utilizing clickstream data to reveal the time management of self-regulated learning in a higher education online learning environment. Interactive Learning Environments.

[B3-behavsci-16-01025] Chen J., Wang R., Fang B., Zuo C. (2024). Fine-grained aspect-based opinion mining on online course reviews for feedback analysis. Interactive Learning Environments.

[B4-behavsci-16-01025] Cristea T., Snijders C., Matzat U., Kleingeld A. (2024). Unobtrusive measurement of self-regulated learning: A clickstream-based multi-dimensional scale. Education and Information Technologies.

[B5-behavsci-16-01025] Dalipi F., Zdravkova K., Ahlgren F. (2021). Sentiment analysis of students’ feedback in MOOCs: A systematic literature review. Frontiers in Artificial Intelligence.

[B6-behavsci-16-01025] Davis F. D. (1989). Perceived usefulness, perceived ease of use, and user acceptance of information technology. MIS Quarterly.

[B7-behavsci-16-01025] D’Mello S., Graesser A. (2012). Dynamics of affective states during complex learning. Learning and Instruction.

[B8-behavsci-16-01025] Ehlert M., Grunschel C., Koehler F. (2025). The role of teachers’ emotions and their assessment in professional development research: A systematic review. Educational Psychology Review.

[B9-behavsci-16-01025] Fleiss J. L. (1971). Measuring nominal scale agreement among many raters. Psychological Bulletin.

[B10-behavsci-16-01025] Gaines R. E., Osman D. J., Maddocks D. L. S., Warner J. R., Freeman J. L., Schallert D. L. (2019). Teachers’ emotional experiences in professional development: Where they come from and what they can mean. Teaching and Teacher Education.

[B11-behavsci-16-01025] Granić A., Marangunić N. (2019). Technology acceptance model in educational context: A systematic literature review. British Journal of Educational Technology.

[B12-behavsci-16-01025] Hargreaves A. (1998). The emotional practice of teaching. Teaching and Teacher Education.

[B13-behavsci-16-01025] Howard N. J. (2021). Barriers and drivers in online micro-course professional development: Navigating issues of teacher identity and agency. Teaching and Teacher Education.

[B14-behavsci-16-01025] Jin F., Song Z., Cheung W. M., Lin C. H., Liu T. (2024). Technological affordances in teachers’ online professional learning communities: A systematic review. Journal of Computer Assisted Learning.

[B15-behavsci-16-01025] Koufakou A. (2024). Deep learning for opinion mining and topic classification of course reviews. Education and Information Technologies.

[B16-behavsci-16-01025] Lantz-Andersson A., Lundin M., Selwyn N. (2018). Twenty years of online teacher communities: A systematic review of formally-organized and informally-developed professional learning groups. Teaching and Teacher Education.

[B17-behavsci-16-01025] Li M., Liu M., Jiang Z., Zhao Z., Zhang J., Ge M., Duan H., Wang Y. (2022). Multimodal emotion recognition and state analysis of classroom video and audio based on deep neural network. Journal of Interconnection Networks.

[B18-behavsci-16-01025] Li Q., Baker R., Warschauer M. (2020). Using clickstream data to measure, understand, and support self-regulated learning in online courses. The Internet and Higher Education.

[B19-behavsci-16-01025] Liu S., Liu S., Liu Z., Peng X., Yang Z. (2022). Automated detection of emotional and cognitive engagement in MOOC discussions to predict learning achievement. Computers & Education.

[B20-behavsci-16-01025] Macià M., García I. (2016). Informal online communities and networks as a source of teacher professional development: A review. Teaching and Teacher Education.

[B21-behavsci-16-01025] Mehrabian A. (1996). Pleasure-arousal-dominance: A general framework for describing and measuring individual differences in temperament. Current Psychology.

[B22-behavsci-16-01025] Meyer A., Kleinknecht M., Richter D. (2023). What makes online professional development effective? The effect of quality characteristics on teachers’ satisfaction and changes in their professional practices. Computers & Education.

[B23-behavsci-16-01025] Mohammadi M., Tajik E., Martinez-Maldonado R., Sadiq S., Tomaszewski W., Khosravi H. (2025). Artificial intelligence in multimodal learning analytics: A systematic literature review. Computers and Education: Artificial Intelligence.

[B24-behavsci-16-01025] Morina F., Fütterer T., Hübner N., Zitzmann S., Fischer C. (2025). Effects of online teacher professional development on teacher-, classroom-, and student-level outcomes: A meta-analysis. Computers & Education.

[B25-behavsci-16-01025] Mu S., Cui M., Huang X. (2020). Multimodal data fusion in learning analytics: A systematic review. Sensors.

[B26-behavsci-16-01025] Mulaimović N., Richter E., Lazarides R., Richter D. (2025). Comparing quality and engagement in face-to-face and online teacher professional development. British Journal of Educational Technology.

[B27-behavsci-16-01025] Nash B. (2022). “We felt like pioneers”: Exploring the social and emotional dimensions of teachers’ learning during online professional development. Journal of Online Learning Research.

[B28-behavsci-16-01025] Pekrun R. (2006). The control-value theory of achievement emotions: Assumptions, corollaries, and implications for educational research and practice. Educational Psychology Review.

[B29-behavsci-16-01025] Pekrun R., Goetz T., Frenzel A. C., Barchfeld P., Perry R. P. (2011). Measuring emotions in students’ learning and performance: The Achievement Emotions Questionnaire (AEQ). Contemporary Educational Psychology.

[B30-behavsci-16-01025] Powell C. G., Bodur Y. (2019). Teachers’ perceptions of an online professional development experience: Implications for a design and implementation framework. Teaching and Teacher Education.

[B31-behavsci-16-01025] Prinsloo P., Slade S., Khalil M. (2023). Multimodal learning analytics—In-between student privacy and encroachment: A systematic review. British Journal of Educational Technology.

[B32-behavsci-16-01025] Russell J. A. (1980). A circumplex model of affect. Journal of Personality and Social Psychology.

[B33-behavsci-16-01025] Samuelsen J., Chen W., Wasson B. (2019). Integrating multiple data sources for learning analytics—Review of literature. Research and Practice in Technology Enhanced Learning.

[B34-behavsci-16-01025] Saunders R. (2013). The role of teacher emotions in change: Experiences, patterns and implications for professional development. Journal of Educational Change.

[B35-behavsci-16-01025] Schneider B. (2024). Three challenges in implementing multimodal learning analytics in real-world learning environments. Learning: Research and Practice.

[B36-behavsci-16-01025] Shaik T., Tao X., Dann C., Xie H., Li Y., Galligan L. (2023). Sentiment analysis and opinion mining on educational data: A survey. Natural Language Processing Journal.

[B37-behavsci-16-01025] Stark K., Bettini E. (2021). Teachers’ perceptions of emotional display rules in schools: A systematic review. Teaching and Teacher Education.

[B38-behavsci-16-01025] Sutton R. E., Wheatley K. F. (2003). Teachers’ emotions and teaching: A review of the literature and directions for future research. Educational Psychology Review.

[B39-behavsci-16-01025] Sümer Ö., Goldberg P., D’Mello S., Gerjets P., Trautwein U., Kasneci E. (2021). Multimodal engagement analysis from facial videos in the classroom. IEEE Transactions on Affective Computing.

[B40-behavsci-16-01025] Taxer J. L., Gross J. J. (2018). Emotion regulation in teachers: The “why” and “how”. Teaching and Teacher Education.

[B41-behavsci-16-01025] Venkatesh V., Davis F. D. (2000). A theoretical extension of the technology acceptance model: Four longitudinal field studies. Management Science.

[B42-behavsci-16-01025] Wang C., Hu X. (2025). Teacher online professional learning: A systematic literature review. Educational Research Review.

[B43-behavsci-16-01025] Yan L., Echeverria V., Jin Y., Fernandez-Nieto G., Zhao L., Li X., Alfredo R., Swiecki Z., Gašević D., Martinez-Maldonado R. (2024). Evidence-based multimodal learning analytics for feedback and reflection in collaborative learning. British Journal of Educational Technology.

[B44-behavsci-16-01025] Yu S., Androsov A., Yan H., Chen Y. (2024). Bridging computer and education sciences: A systematic review of automated emotion recognition in online learning environments. Computers & Education.

